# The length of the biliopancreatic limb in one anastomosis gastric bypass

**DOI:** 10.3389/fsurg.2024.1248744

**Published:** 2024-08-14

**Authors:** Marc Focquet

**Affiliations:** Bariatric and Metabolic Surgery Unit, Department of General and Abdominal Surgery, AZ Sint Elisabeth Hospital, Zottegem, Belgium

**Keywords:** biliopancreatic limb length (BPLL), total small bowel length (TSBL), common limb length (CLL), one anastomosis gastric bypass (OAGB), weight loss surgery, obesity

## Abstract

**Introduction:**

The one-anastomosis gastric bypass (OAGB), first published by Dr Rutledge in 1997 is now a well-established procedure in the bariatric-metabolic armamentarium. This procedure based on a (single) loop gastro-jejunal anastomosis (the biliopancreatic limb or BPL) with a long narrow gastric pouch combines restriction with hypo-absorption. The biliopancreatic limb and in particular its length is held responsible for the degree of the hypo-absorptive effect but the most appropriate or “optimal” length of the BPL remains debatable.

**Methods:**

The following text is based on a comprehensive and meticulous selection of the most recent literature in Cochrane, Pubmed and Google Scholar using the search terms “biliopancreatic limb”, "biliopancreatic limb in one anastomosis gastric bypass” in an attempt to define not only the most common used biliopancreatic limb length but also to find out If there is an “ideal” limb length not only to optimize the outcomes of the OAGB in terms of weight loss and resolution of obesity-related diseases but also to reduce the potential side-effects in particular nutritional deficiencies.

**Results:**

Until today there is no consensus about the “standard” or “ideal” length of the biliopancreatic limb in OAGB, a fixed length of 200 cm is still the most common used procedure although many reports and studies are in favour of shorter limb lengths adjusted to the BMI or the total small bowel length.

**Conclusion:**

The “ideal” or “optimal” biliopancreatic limb length in OAGB still needs to be defined. There are different options and all of them have their credits, the question remains if a consensus can be reached regarding the best strategy to obtain the best outcome.

## 1 Introduction

Obesity has become a real global pandemic responsible for severe associated diseases as there are diabetes type 2, hypertension, sleep apnea, arthritis, hyperlipidemie and others so its treatment is of paramount importance. Among the different treatments (diets, medication, physical activity, counseling, psychotherapy), weight loss surgery is probably the most effective therapy at this moment. The potential mechanisms and physiology of weight loss surgery are multifactorial and not fully elucidated: age, genetics, changes in gut microbiota, alterations in bile, gut hormones (GLP1, PYY), food restriction and hypo-absorption are some of the key- factors. Today dysfunctional neuroendocrine signals are held responsible for an impaired ingestion resulting in overweight and obesity so bariatric surgery has become bariatric-metabolic surgery if not purely “metabolic surgery” (1). In this whole process the biliopancreatic limb and in particular its length plays a crucial role when it comes to weight loss in bypass surgery.

## 2 The “one fits all” biliopancreatic limb length

### 2.1 The early days

**Figure 1:**
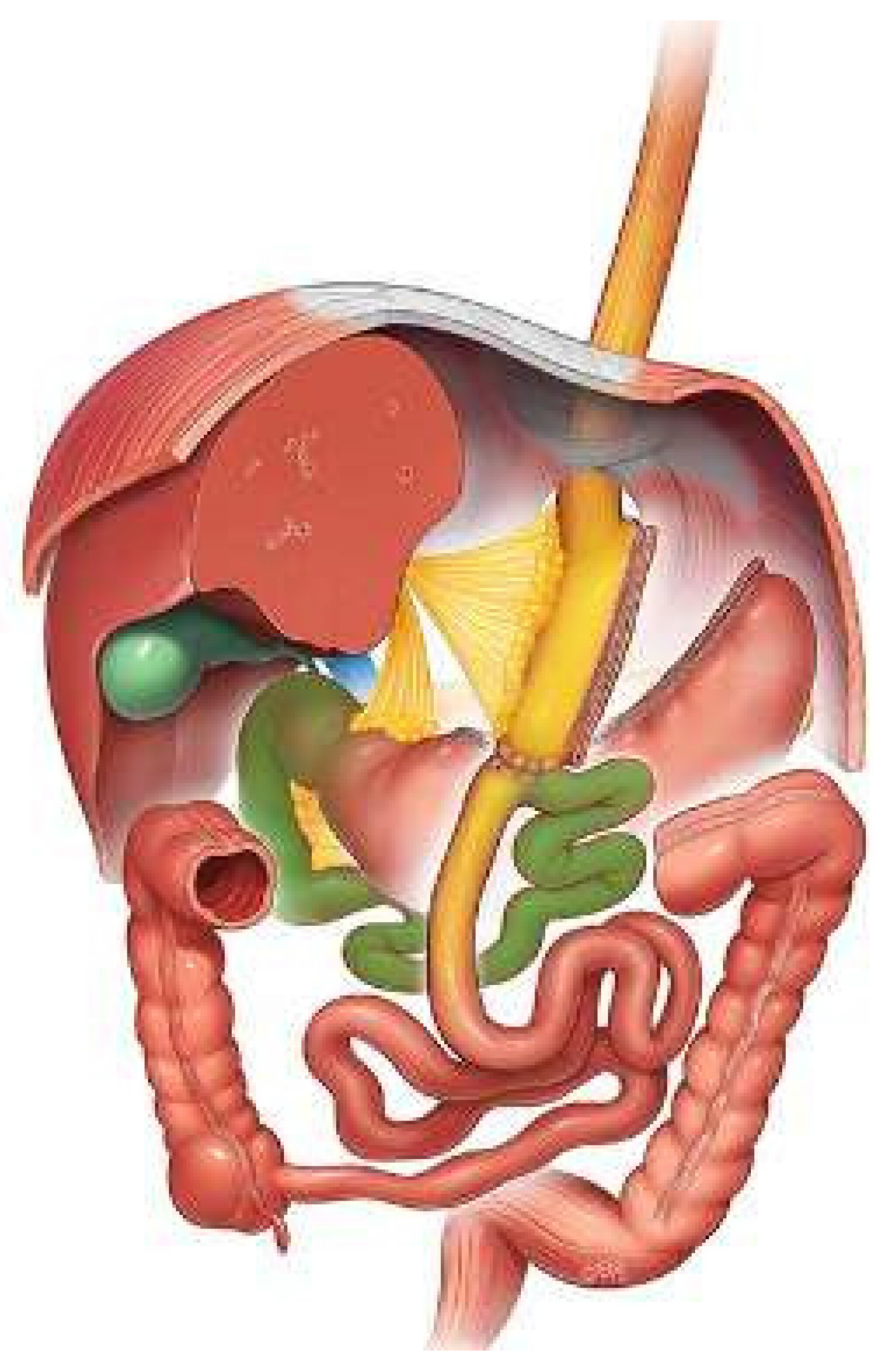
One anastomosis gastric bypass: long gastric pouch (yellow), biliopancreatic limb (green).

Dr Robert Rutledge—“inventor” of the Mini Gastric Bypass (MGB) or One Anastomosis Gastric Bypass (OAGB) (Figure 1)—in his early publications (2, 3) proposed a BPL length of 180 cm–200 cm. In his article “**The Mini-Gastric Bypass original technique**” (4) Rutledge states that the correct form of the BPL is a critical factor in the success of the operation and has a length of **1.5**–**2 m** distal to the ligament of Treitz. Also Wei-Jei Lee used a BPLL of 200 cm in his early experience (5). In his meta-analysis in 2013 “**Mini Gastric Bypass: systematic review of a controversial procedure**” Kamal Mahawar (6) noticed that the most used BPL length was 200 cm. Another meta-analysis by Solouki and Kermansaravi in 2018 (7) confirmed the use of a fixed length between 150 and 200 cm.

### 2.2 Towards shorter limb lengths…

Because of an increasing concern of protein-calorie malnutrition with the 200 cm BPLL, as described by Khalaj (8) (who described 7 patients with PCM in a series of 189 patients after OAGB with 200 cm BPLL) the opinion to shorten the “fixed” BPLL to a length of 150 cm gained more interest. In 2019 Neuberg et al. (9) published the first 5-year follow-up study with a 150 cm BPLL in 163 patients with the following conclusions: “**OAGB with a 150 cm BPLL is safe and effective for weight loss and reduction of co-morbidities and patients are satisfied with their quality of life (QoL)**”. A retrospective study of 343 patients with a follow-up of 18–24 months by Boyle and Mahawar (10) showed similar weight loss and resolution of obesity-associated diseases with a 150 cm BPL compared to a 200 cm BPLL even in super-obese patients (BMI > 50). This was confirmed by Liagre et al. (11) in a review of 245 patients with a BMI > 50, published in 2021. In his retrospective matched cohort study Bertrand (12) did not observe a significant difference in mean %EBMIL between a BPLL of 150 cm vs. a BPLL of 200 cm but hypoalbuminemia, Vitamin B and ferritin deficiencies were more pronounced in the 200 cm group. An 8-year follow-up study of OAGB with a 150 cm BPLL by Liagre (13) showed good long-term outcomes regarding weight loss, quality of life and resolution of obesity-associated diseases with a very low rate of protein-calorie malnutrition. When comparing the 10-year outcome of a 150 cm BPLL OAGB with Roux-n-Y gastric bypass the previous author (14) found similar results: both procedures are effective but OAGB is associated with shorter operative times and better results in short- and long-term morbidity and weight loss outcomes. Piazza (15) also compared BPLL of 150 cm, 180 cm and 200 cm and after a 2-year follow-up he could not found a significant difference in weight loss nor in resolution of obesity-associated diseases and nutritional deficiencies except for iron and ferritin. An Australian study of 325 OAGB procedures by Gricks (16) with a fixed BPPL of 150 cm reported also excellent WL after 1 year FU (Table 1).

**Table 1:** OAGB with a fixed biliopancreatic limb length.

Author	Year	Number of pts	Follow up	Mean BMI	BPLL	% EWL
Rutledge	2001	1,274	24 months	47	200 cm	77%
Rutledge	2005	2,410	38 months	46	200 cm	80%
Wei-Jei-Lee	2005	423	36 months	44.2	200 cm	70.5%
Hussain	2018	519	36 months	48	150 cm	77%
Boyle	2019	118	18–24 months	47.8	150 cm	74%
		225	18–24 months	49.7	200 cm	75%
Piazza	2020	52	24 months	44	150 cm	60.7%
		53	24 months	43	180 cm	61.6%
		52	24 months	44	200 cm	61.2%
Neuberg	2020	163	60 months	41.2	150 cm	81.8%
Liagre	2020	115	96 months	43.2	150 cm	84.8%
Liagre	2021	245	80 months	54	150 cm	80.5%
Gricks	2022	325	12 months	43.3	150 cm	74 %

### 2.3 Systematic reviews and meta-analysis…

Parmar (17) in his **systematic review** of OAGB as a metabolic operation in patients with a BMI < 35 reported satisfying results with a median BPLL of 120 cm. In conclusion of his **meta-analysis** “Effect of biliopancreatic limb length on weight loss, postoperative complications, and remission of co- morbidities in OAGB” Tasdighi (18) suggests standardization of BPLL **shorter than 200 cm** because a bypass length of 200 cm or more did not increase weight loss but was related to more frequent complications and nutritional problems. In a **systematic review and meta-analysis** analysing the 150 cm BPLL OAGB compared to a 200 cm BPLL, published in 2023, Salman (19) found that a BPLL of 200 cm is still the most commonly used limb length followed by a better weight loss outcome than a 150 cm BPLL but at the expense of more nutritional deficiencies. Remission of obesity-associated diseases was similar in both groups.

**Discussion:**

With growing experience and an increasing number of studies and reports a lot of surgeons switched from the “historical” 200 cm to the shorter fixed BPLL of 150 cm. An even shorter BPLL of 120 cm or less could be used for patients with obesity class 1 with metabolic disorders (17). In an animal model Ribeiro-Parenti et al. (20) demonstrated that “shortening the BPLL allows similar positive outcomes on enterohormone (GLP1, PPY) secretion and glucose metabolism”. Mahawar (21) in his “letter to the editor” in Obesity Surgery in 2021 suggests even to study a BPLL of 100 cm.

The BPLL of 150 cm is safe and followed by good weight loss and resolution of obesity-related diseases without causing nutritional deficiencies. Surprisingly despite the numerous reports and studies in favour of a 150 cm BPLL, the most commonly used BPLL still is 200 cm (19). But Hussain (22) in his publication of 519 primary OAGB with 150 cm BPLL in patients with BMI < 50 and 200 cm for BMI > 50 reported 2 patients with severe liver dysfunction (in both patients BPLL was >200 cm) whereupon he addressed the following warning: “never do BPLL more than 200 cm”! Elgeidi et al. (23) in their large serie of 692 OAGB patients with a BPLL of 200 cm and < 200 cm reported protein-energy malnutrition in 2,3%. During revisional surgery and measurement of the bowel lengths they found in all cases a common channel of at least 300 cm!

Key points:

➢ A fixed BPLL of 150 cm is a safe option and results in good weight loss and resolution of obesity-associated diseases without nutritional deficiencies (9, 10–12).
➢ A BPLL of 200 cm can result in higher weight loss although not statistically significant but at the expense of nutritional deficiencies (19, 23).
➢ A BPLL of 200 cm is still the most used “fixed” length.

## 3 The biliopancreatic limb length tailored in relation to the BMI

### 3.1 The concept…

The concept of tailoring the BPLL was introduced by Prof Wei-Jei Lee (24) and first published in 2008. It was based on his opinion that a fixed BPPL of 200 cm might not be appropriate for patients with lower BMI's or an extreme high BMI. So he used a BPLL of 150 cm for BMI 35 with a 10 cm increase in the bypass length with every BMI point, resulting in a mean bypass length of 150 cm, 250 cm and 350 cm for the lower, medium and higher BMI categories. In his series of 644 patients the weight loss was satisfying and besides a statistically significant lower haemoglobin in the lower BMI group he did not noticed relevant nutritional deficiencies.

### 3.2 The studies and reports…

Noun (25) in his clinical report “**One thousand consecutive Mini Gastric Bypasses: short-and long term outcomes**” used a BPLL of 150 cm increased by 10 cm for each BMI point above 40 and found similar good weight loss as described in the study by Wei-Jei- Lee. He noticed excessive weight loss and malnutrition in only 4 patients all within the lower BMI group (BMI 35 or <). In the Italian experience of 974 consecutive cases reported by Musella et al. (26) the BPPL was tailored according to Lee with a mean length of 224.6 cm ± 23.2 cm, and besides excellent weight loss with a EWL% of 77% after 5 years (comparable with the results of Lee and Noun) they found excessive weight loss in 2 patients and iron deficiency anemia in 5.3% of the patients. Taha et al. (27) analysed the outcomes of OAGB in 1520 patients in a 6-year FU with a BPLL based on the BMI with a range from 150 cm to 300 cm. He reported excellent weight loss (80% EWL), iron deficiency was 3.1% and excessive weight loss (EWL > 100%) occurred in only 3 patients. Adjusting BPLL to the BMI and patient's age in a retrospective cohort study of 653 patients by Kermansaravi (28) resulted also in good to excellent outcomes in terms of weight loss with minimal complications.

### 3.3 However…

Jain et al. (29) in a subgroup analysis of a randomized control trial, comparing OAGB to sleeve gastrectomy with a BPLL of 150 cm and 180 cm tailored to the BMI and the presence of diabetes, did not found any significant difference in %EWL between the 2 groups. He concluded that BPLL of 150 cm may be sufficient in all OAGB patients. In a retrospective cohort study Slagter et al. (30) used a BPLL of 150 cm for BMI < 40, 180 cm for BMI 40–44.9 and 200 cm for BMI 45–49.9. Their data analysis showed no significant differences in weight loss. Charalampos (31) in a limited study of 94 patients with tailored BPLL of 200 cm, 250 cm and 300 cm, according to BMI < 50, 50–60 and >60 found similar results: no significant difference in weight loss nor in the incidence of nutritional deficiencies (Table 2).

**Table 2:** OAGB with biliopancreatic limb length tailored to BMI.

Author	Year	Number pts	Follow up	BMI	BPLL	% EWL
Wei –Jei-Lee	2008	644	24 months	<40	150 cm	79.1%
				40–50	250 cm	73.1%
				>50	350 cm	67.2%
Noun	2012	1,000	60 months	42 (mean)	150 cm	68.6%
					+10 cm/BMI	Point>40
Musella	2014	974	60 months	48 (mean)	224 cm (mean)	77%
Taha	2017	1,520	36 months	46.8 (mean)	150–300 cm	80.2%
Charalampos	2019	94	36 months	<50	200 cm	98.7%
				50–60	250 cm	84.3%
				>60	300 cm	80.2%
Kermansaravi	2020	653	12 months	35–39	180 cm	96%
				40–50	200 cm	84%
				>50	220 cm	68%
Slagter	2021	632	36 months	<40	150 cm	83%
				40–44.9	180	77%
				45–49.9	200 cm	75%
Jain	2022	63	60 months	39.73	150 cm	64.2%
		38	60 months	51.92	180 cm	66.5%

**Discussion:**

A biliopancreatic limb length (BPLL) based on the patient's BMI did not result in statistically significant higher percentage excess weight loss (%EWL) for patients with preoperative higher BMI, but it can be an option to consider in high BMI's. It was the onset to tailor or adjust the biliopancreatic limb.

Key points

➢ The BPLL can be tailored in relation to the BMI.
➢ No significant difference in weight loss was found in comparison with the “fixed” 150 cm BPLL.

## 4 The biliopancreatic limb length tailored in relation to the total small bowel length

### 4.1 The total small bowel length

The measurement of the limb lengths in intestinal bypass procedures can be of utmost importance for a successful outcome not only in weight loss but also in the prevention of protein-calorie malnutrition (32). The mean length of the small bowel reported by Tacchino (33) was 690 cm with a standard deviation of ±93.7 cm. To notice that 15% of males had a TSBL longer than 800 cm and 5% of the measurements were shorter than 400 cm! This was confirmed in the study by Purandare (34) and also by Bekheit (35) who published the largest series of bowel measurement with 606 participants with a mean TSBL of 630 cm ± 175 cm and a range that varies from 250 cm to 1300 cm!

### 4.2 The BAGUA

Carbajo (36) was the first surgeon to emphasize the importance of the relation BPLL/TSBL and CLL/TSBL in his search for “optimal” weight loss (BMI 25) and complete remission of obesity-assiciated diseases without disturbing the nutritional balance. The technique that he developed together with Garcia-Caballero, was named **BAGUA** (Bypass Gastrico de Una Anastomosis) or “One Anastomosis Gastric Bypass” in English.The concept was based on the original MGB technique, but was different in defining the biliopancreatic limb length and also a particular anti-biliary-reflux procedure was part of his procedure. Although in his initial series published in 2005 he used a fixed BPPL of 200 cm (37) he soon started to measure the total small bowel length to determine the appropriate BPLL and CLL and he selected the mid-portion of the total bowel as the bypassed length and for higher BMI he added 10–50 cm but always maintained at least 250 cm of CLL. It should be noted that the mean length of the TSBL in his series was not longer than 500 cm!

In the 5-year follow up study of 350 consecutive patients with primary OAGB, published in 2019 Ruiz-Tovar and Carbajo (38) concluded that the best and safest method to obtain optimal outcomes in weight loss and resolution of co-morbidities was to quantify the total small bowel length to define the “optimal” BPLL and CLL: the length of the common channel and the relation CLL/TSBL seemed to be the most accurate parameters in order to achieve the best weight loss (BMI 25) and the CLL/TSBL ratio of 0,4–0,43 delivered the best outcomes.

### 4.3 Variation on the same theme….

Komaei et al. (39) compared a group of patients with a fixed BPLL of 200 cm to a “tailored group” with a BPLL of 40% of the TSBL in a 1-year follow-up and decided that tailoring the BPLL was “even likely superior to the fixed 200 cm BPL with less nutritional deficiencies”. Abdallah (40) came to similar results when he compared a 200 cm fixed BPL with a tailored BPLL (proximal 1/3 of the TSBL). He found greater weight loss and similar improvement of co-morbidities and less nutritional (hypoalbuminemia) problems in the tailored group. These findings were confirmed by a prospective randomized control trial with a fixed BPLL of 200 cm and a tailored BPLL of one third of the TSBL in a 1-year follow up reported by Zaki (41). Despite a small sample of patients (30 in each group) he found a wide range of TSBL (420 cm–920 cm). Another tailoring formula was applied by Ruiz-Mar (42) with a BPLL of 30% TSBL for patients with BMI 35–50, 35% for patients with any degree of diabetes and 40% for super obese patients (BMI > 50), he noticed good weight loss and metabolic improvement in a 1-year follow up of 51 patients (Table 3).

**Table 3:** Biliopancreatic limb length with measurement of the total small bowel length.

Author	Year	Number pts	Follow-up	BMI	BPLL	CLL	%EWL
Carbajo	2017	1,200	12 years	46	50% TSBL	50% TSBL	70%
Ruiz-Tovar	2019	350	5 years	41.3	57%–60% TSBL	40%–43% TSBL	94%
					(formula: CLL/TSBL: 0.4–0.43)	
Komaei	2019	32	1 year	45	40%	60% TSBL	63.3%
Ruiz-Mar	2019	51	1 year	48.2	30%–40%TSBL	70–60% TSBL	65%
Abdallah	2020	40	1 year	49.7	1/3 TSBL	2/3 TSBL	80.2%
Zaki	2022	30	1 year	56.5	1/3 TSBL	2/3 TSBL	76.1%

In a 2-year follow-up study of 214 patients with BMI > 50 published in 2022 by Eskanderos (43) the TSBL was measured to ensure a CLL of at least 350 cm. In his conclusion this author states that “**OAGB in patients with a BMI > 50 and a total bowel length >6 m can achieve a target BMI of 25 with a 40:60% relation BPLL/CLL**”.

A large randomized control trial (Tailor study) was started in 2020 in the Netherlands by the group of Slagter et al. (44), the first large RCT concerning a BPLL tailored to the TSBL with a 150 cm BPLL for TSBL < 500 cm, 180 cm for TSBL 500–700 cm, 210 cm for TSBL > 700 cm vs. a fixed BPLL of 150 cm with 106 patients in each arm. Primary endpoint will be the percentage total weight loss (% TWL) after a 5-year follow up. Secondary endpoints are % TWL at different times, remission of associated diseases, nutritional deficiencies, quality of life, defecation and dumping.

### 4.4 Consensus?…..

A consensus (79% agrees) was reached in the IFSO consensus conference statement on OAGB in 2020 (45) on the statement: “measurement of the total bowel length can be used to define the percentage for the length of the biliopancreatic limb length” but on the statement “total bowel should always be measured for safe and adequate OAGB” the experts could not reach a consensus (59% disagrees). Out of 742 responders of the IFSO Worldwide One Anastomosis Gastric Bypass Survey in 2020 (46), only one third measured the TSBL and the **most common used length was 200 cm**. The tailored approach was used by 27% of the surgeons.

**Discussion:**

There is a wide variety of small bowel lengths in humans and unfortunately there is no clear relationship between TSBL and BMI, age and sex, besides a weak link with height. As demonstrated by Carbajo (38) measurement of the TSBL permits to calculate the appropriate ratio (CLL/TSBL: 0.40–0.43) for optimal weight loss (BMI 25) with excellent remission of co-morbidities it also reduces the risk of nutritional deficiencies. Measurement of the TSBL can help to improve not only the efficacy but also the safety of OAGB. More studies and RCT's are needed to reach a consensus.

Key points:

➢ There is a wide variation in the total small bowel length.
➢ Tailoring the bypassed length in relation to the total bowel length can improve the efficacy and safety of the OAGB procedure.
➢ There is no consensus on the systematic measurement of the TSBL.

## 5 The fixed common limb length

In 2013 Radwan Kassir (47) developed what he called the OLGIBP (omega loop gastroileal bypass) with a fixed length of the common channel of 300 cm. In his limited patient cohort, published in 2021, he found excellent weight loss similar to the “standard” 200 cm BPLL OAGB, no malnutrition and less bile reflux in a 3-year follow up. The same procedure was described by De Luca et al. (48) in an article published in Obesity Surgery in 2017 entitled “A New Concept in Bariatric Surgery. Single Anastomosis Gastro-Ileal Bypass (SAGI): Technical details and Preliminary Results”: a modification of the OAGB operation based on the SADI-S concept, with a CLL of 300 cm and a gastro-ileal anastomosis instead of a duodeno-ileal anastomosis as in SADI. With only a limited number of 7 patients and a short follow up of 3–6 months, they reported excellent weight loss, without bile reflux and no nutritional complications (no anemia nor hypo proteinemia).

A more consistent study was done by Nabil et al. (49) in a randomized controlled trial with a 1-year follow up. In two groups of 30 patients they compared the conventional OAGB with a BPLL of 200 cm to a fixed CLL of 400 cm. No significant difference in weight loss was found but haemoglobin, total protein and albumin levels were significant lower in the fixed CLL group. In both groups the TSBL was very similar with a mean length of 720 cm (range 600–1000 cm). Soong (50) compared the BPLL tailored to the BMI with a fixed CLL of 400 cm after measurement of the TSBL and the results of the two groups regarding weight loss and remission of co-morbidities were similar.

Also the “Ileal food diversion” developed by Greco and Tacchino (51) can be considered as a modified OAGB with a fixed CLL of 300 cm but without dissection of the gastric fundus. In their consecutive series of 68 patients with a mean follow up of 9 months they reported excellent weight loss and resolution of co-morbidities, 2 patients had inadequate weight loss and 1 patient developed protein-malnutrition (Table 4).

**Table 4:** OAGB with fixed common channel length.

Author	Year	Number pts	Follow-up	BMI	CLL	Weight loss
Greco	2015	68	24 months	43.5	300 cm	89% EWL
De Luca	2017	7	6 months	42.1	300 cm	82.1% EWL
Nabil	2019	30	12 months	54.9	400 cm	69.4% EWL
Kassir	2021	17	36 months	45.3	300 cm	48.2% TWL

**Discussion:**

The concept of a fixed common channel length in OAGB results in good to excellent weight loss but measurement of the total small bowel length seems mandatory to reach the provided goals and to avoid nutritional complications.

Key points:

➢ A fixed CCL can be an alternative to a fixed or tailored BPLL.
➢ Length of the common channel has to be at least 300 cm.
➢ Measurement of the TSBL is recommended to avoid nutritional deficiencies.

## 6 Conclusion

At this moment there is no consensus concerning the most appropriate biliopancreatic limb length. Obviously according to the results of systematic reviews (6, 7, 19), and the worldwide IFSO survey (46) the 200 cm BPLL is still the most used length in OAGB despite the similar outcomes with a 150 cm BPLL (10–12) and despite the increased risk of malabsorption (12, 23). The IFSO consensus meeting (45), with a panel of 52 recognized OAGB experts from 28 countries reached a clear consensus that a biliopancreatic limb length should be equal or less than 200 cm and that the total small bowel length has to be measured for longer BPLL. Consensus was also reached concerning the use of a fixed length and tailoring the length according to the BMI. Routine measurement of the total small bowel was however not accepted as standard practice. In conclusion we can state that all options concerning the length of the biliopancreatic limb have deserved credits and can be applied when performing a one anastomosis gastric bypass. Several factors can play a role in the choice of the preferred BPLL: routine, personal experience,safety, efficacy, scientific evidence, patient's compliance, follow-up…., there are no bad options as long as they comply with good common practice taking into account the actual scientific data. The “ ideal” or “ optimal “ biliopancreatic limb length still needs to be defined and the question remains if we will ever come to the “ gold standard “ BPLL resulting in the “ perfect “ OAGB. Only the future will tell.
